# Acid-sensing ion channels: trafficking and synaptic function

**DOI:** 10.1186/1756-6606-6-1

**Published:** 2013-01-02

**Authors:** Xiang-ming Zha

**Affiliations:** 1 Department of Cell Biology and Neuroscience, College of Medicine, University of South Alabama, 307 University Blvd, MSB1201, Mobile, AL, 36688, USA

**Keywords:** Acid-sensing ion channel (ASIC), Acidosis, Calcium, Dendritic spine, Glycosylation, Signaling, Synapse, Trafficking

## Abstract

Extracellular acidification occurs in the brain with elevated neural activity, increased metabolism, and neuronal injury. This reduction in pH can have profound effects on brain function because pH regulates essentially every single biochemical reaction. Therefore, it is not surprising to see that Nature evolves a family of proteins, the acid-sensing ion channels (ASICs), to sense extracellular pH reduction. ASICs are proton-gated cation channels that are mainly expressed in the nervous system. In recent years, a growing body of literature has shown that acidosis, through activating ASICs, contributes to multiple diseases, including ischemia, multiple sclerosis, and seizures. In addition, ASICs play a key role in fear and anxiety related psychiatric disorders. Several recent reviews have summarized the importance and therapeutic potential of ASICs in neurological diseases, as well as the structure-function relationship of ASICs. However, there is little focused coverage on either the basic biology of ASICs or their contribution to neural plasticity. This review will center on these topics, with an emphasis on the synaptic role of ASICs and molecular mechanisms regulating the spatial distribution and function of these ion channels.

## Introduction

Acid-sensing ion channels (ASICs) are a family of proton-gated cation channels which are expressed primarily in the nervous system [1,2]. ASICs belong to a superfamily which also contains degenerins (DEG) and epithelial sodium channels (ENaC). All members within the ENaC/DEG/ASIC (EDA) superfamily share the same topology, with the N- and C-termini located inside the cell and a large well organized, cysteine-rich extracellular domain [1,2]. There are four genes encoding at least six different ASICs (Table 1). ASICs function as trimers [3,4] and conduct mostly Na^+^. Homomeric ASIC1a and human ASIC1b, as well as ASIC1a/2b heteromers, also have a low permeability to Ca^2+^[5-9]. Interestingly, at pH 7.25 (but not at pH 5.5), a complex between a truncated chick ASIC1 and Psalmotoxin (PcTx1) is non-selective to monovalent cations [10]. This result indicates that ion selectivity of ASICs can be altered under specific conditions. ASIC1 and ASIC3 start to open at about pH 7 and 7.2, respectively, which enables them to sense pH changes within physiological pH ranges [5,11-15]. In addition, a recent study shows that human ASIC3 can also respond to alkanization [16]. Since extracellular pH fluctuation is common in pathological conditions, it is conceivable that ASICs play important roles in a wide spectrum of neurological diseases. Consistent with this speculation, ASICs contribute to pain, brain ischemia, multiple sclerosis, seizures as well as anxiety-related disorders [1,2,17,18]. These findings highlight the potential therapeutic value of targeting ASICs in diseases. In addition, recent studies reveal that ASICs and acidosis regulate dendritic spines, the site of most excitatory neurotransmission in the brain [19-21]. These data suggest that ASICs also play an important role in regulating neural plasticity in pathological conditions.

**Table 1:** Basic channel properties, activators and inhibitors of ASICs

**Gene**	**Protein**	**pH**_**50**_	**Agonist (EC**_**50**_**)**	**Antagonist (IC**_**50**_**)**	**Ions passed**	**References**
*ASIC1* *(ACCN2)*	ASIC1a	6.2–6.8	MitTx (9.4 nM)	PcTx1 (~1 nM)	Na^+^ > Ca^2+^	[5,12,13,22-27]
				Mambalgin (55 nM)		
				Sevanol (2.2 mM)		
				A-317567 (2 μM)		
	ASIC1b	~6.0 (mouse)	MitTx (23 nM)	Mambalgin (192 nM)	mouse: Na^+^ human: Na^+^ > Ca^2+^	[6,7,12,13,22,24,28]
		~6.2 (human)				
*ASIC2 (ACCN1)*	ASIC2a	4.5–4.9	Minimal activa-tion by MitTx	A-317567 (29 μM)	Na^+^	[12,15,22,25,29-31]
	ASIC2b	N/A			--	[29-31]
*ASIC3*	ASIC3	~6.6	GMQ (0.35 mM)	APETx2 (63 nM)	Na^+^	[13,22,25,32-36]
			MitTx (830 nM)	Sevanol (0.35 mM)		
				A-317567 (9.5 μM)		
*ASIC4*	ASIC4	N/A			--	[37]
Heteromeric channels	1a + 1b	6.0		Mambalgin (72 nM)	Na^+^	[15]
	1a + 2a	5.5–6.1		Mambalgin (246 nM)	Na^+^	[12,15,24,26]
	1a + 2b	Same as ASIC1a		Mambalgin (61 nM) PcTx1 (~3 nM)	Na^+^ > Ca^2+^	[9,15,24]
	1a + 3	6.3–6,6			Na^+^	[12,15]
	1b + 3	6.0			Na^+^	[15]
	1b + 2a	4.9			Na^+^	[15]
	2a + 3	5.7–6.1			Na^+^	[12,15]
	2b + 3	6.5		APETx2 (117 nM)	Na^+^	[15,33,36]

Note: amiloride is not included in the table because it functions as a universal blocker for ASICs and many other ion channels, such as ENaCs and sodium/hydrogen exchangers.

### Recent advances in ASIC pharmacology

Although the canonical ligand for ASICs is protons, the massive extracellular domain of ASICs has led to the speculation that these receptors may also respond to other ligands [38]. Supporting this speculation, ASIC channels are potentiated by FMRFmide like peptides, dynorphin A and big dynorphin; all reduce the steady-state desensitization of ASICs [39-41]. In addition, spermine, a polyamine that is released in injury conditions, also potentiates ASIC currents by reducing the steady-state desensitization [11,42]. These studies, along with the link between ASICs and multiple diseases, have inspired the screening for novel ASIC ligands/antagonists using various approaches. The first non-proton ligand identified is 2-guanidine-4-methylquinazoline (GMQ), which activates ASIC3 at normal pH [32]. In addition, Bohlen et al. recently isolated a peptide, MitTx, from the venom of Texas coral snake [22]. At neutral pH, MitTx induces sustained opening of ASICs, and is highly selective to ASIC1 (see Table 1). These findings not only provide novel tools for studying ASIC function but also suggest that physiological ligands other than protons exist.

Various ASIC inhibitors have also been isolated from venoms. PcTx1, a toxin isolated from the venom of tarantula spider, is specific to ASIC1a homomeric and ASIC1a/2b heteromeric channels [9,23]. APETx2, on the other hand, inhibits ASIC3 containing channels [33]. Recently, another class of peptides, named mambalgins, were isolated from black mamba venom; mambalgins inhibit several ASICs and abolish pain sensation [24]. Besides these peptides, several small molecules also inhibit ASICs. One commonly used small molecule inhibitor is amiloride. Though unspecific, amiloride has the advantage in that it has been used clinically for decades. A-317567 is a synthetic compound which inhibits ASIC1a, 2a and 3 [25]. Sevanol, or epiphyllic acid 9, 10-diisocitryl ester, isolated from a plant, *Thymus armeniacus*, inhibits both ASIC3 and ASIC1a, with no effect on other ASIC subunits [34]. A summary of these ASIC activators and inhibitors is presented in Table 1. These reagents are useful for manipulating ASIC activities in functional studies. However, the development of additional subunit-specific pharmacological inhibitors will be valuable for targeting of ASICs in diseases.

### Expression and subcellular localization of ASICs

#### Overall expression of ASICs

The distribution of ASIC mRNA is wide spread at both peripheral and central nervous systems (PNS and CNS). The PNS expresses all ASIC subunits [5,7,29-31,35,43]. The CNS, on the other hand, primarily expresses ASIC1a, ASIC2a, and ASIC2b [29-31,35]. Almost all neurons possess robust acid-evoked ASIC type current [44-47]. Other than neurons, NG2 glia cells [48], some receptor cells [49-52], vascular smooth muscle [53], and immune cells [54] also express ASICs, though typically at lower levels. In addition, many types of brain tumors upregulate ASIC expression [55,56], which suggests a possible role of ASICs in tumor physiology.

Consistent with the *in situ* and PCR results, western blot and immunostaining detect ASIC1a protein in most brain regions. However, there are variations in the relative expression level of ASIC1a among different structures. ASIC1a levels are higher in amygdala, cingulate, periaqueductal gray, layer III of somatosensory cortex and striatum [57-59]. Within the hippocampus, the dentate gyrus region shows higher ASIC1 immunostaining than CA1 and CA3 [59]. Detailed localization of endogenous ASIC2 protein is not yet available, largely due to the lack of a reliable ASIC2 antibody for detecting endogenous ASIC2 proteins [20,52]. However, most of the currents recorded from CNS neurons show a mixed contribution from ASIC1a homomeric, ASIC1a/2a, and ASIC1a/2b heteromeric channels [9,45,46,58,60-62]. In addition, the properties of acid-activated current recorded suggest that the relative expression of ASIC2 varies among different brain regions. For example, currents in medium spiny neurons in striatum contain a predominant ASIC1a homomeric component [61]. Similarly, cerebellar Purkinje cells exhibit currents representative of ASIC1a homomers or 1a/2b heteromers [60]. In hippocampus, the basket cells, one class of interneuron, expresses primarily ASIC1a while many other interneurons express both ASIC1a and ASIC2 [47].

Besides the variation among brain regions and cell types, there may also be developmental changes in ASIC expression. In one study, increased maturation of the neuronal culture is associated with increased ASIC2:ASIC1a mRNA ratio and reduced inhibition of ASIC currents by PcTx1 [63], suggesting that the relative ASIC2 protein levels are increased with maturation/aging. There may also exist species differences. In cultured human cortical neurons, acid-activated currents appear to come predominantly from ASIC1a homomers [64]. In contrast, currents in mouse/rat neurons typically exhibit a higher contribution from ASIC1a/ASIC2 heteromers. This difference is interesting. However, in the human study, neurons were isolated from patients of 23–72 years old and recorded at 3–4 days in culture [64]. Most rodent studies, on the other hand, were performed on neurons obtained from embryonic or early postnatal animals and cultured for 7–21 days. Therefore, caution shall be taken as to whether the differences observed are completely attributable to species. Nevertheless, these results indicate that it will be important to study the biogenesis and function of human ASICs, preferably in human neurons.

#### Subcellular localization of ASICs in neurons

In peripheral neurons, ASIC1, 2, and 3 are detected in axons, axon terminals and cell bodies [43,65,66]. In the brain, both ASIC1a and ASIC2a show a preferential somatodendritic distribution [67-69]. In cultured CNS neurons, endogenous ASIC1a localizes primarily to dendrites with little detectable presence in axons [19,44,70]. Similar distribution is observed when ASIC1a and ASIC2a are expressed in organotypic hippocampal slices [19-21,71]. Of note, one previous study reports a ubiquitous distribution of ASIC1 in cultured brain neurons [57]. The reason for this discrepancy is unclear, although it is noted that the antibodies used in the above studies are different. The healthiness of the neuron also regulates ASIC localization. In spinal cord, endogenous ASIC1a is barely detectable in healthy axons, but increases drastically when the axons are injured [72]. Taken together, these data demonstrate a preferential somatodendritic targeting of ASIC1a in CNS, and suggests that altered subcellular traffficking of ASICs plays a critical role in pathological conditions.

**Figure 1:**
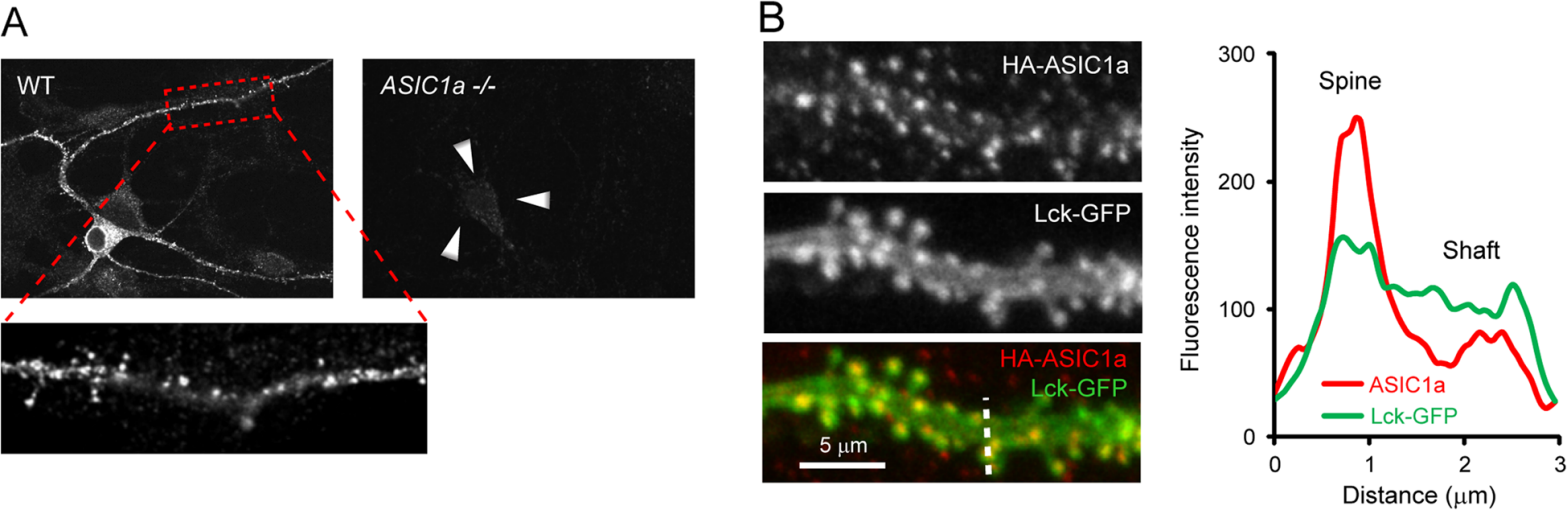
**ASIC1a localization in dendritic spines. A**) Immunofluorescence images showing the localization of endogenous ASIC1a in cultured neurons. Wild type (WT) and *ASIC1a−/−* cortical neurons were stained with an anti-ASIC1a antibody. Inset at the bottom shows a high magnification view of the boxed region in WT. Note the punctate staining along the dendrite, suggesting the presence of ASIC1a in dendritic spines. **B**) Confocal images showing the enrichment of ASIC1a in spines of slice neurons. Organotypic hippocampal slices were transfected with HA-ASIC1a and a membrane-targeted Lck-GFP. ASIC1a localization was detected by immunostaining with an anti-HA antibody. Plot on the right shows the relative fluorescence intensity of ASIC1a and Lck-GFP along the line drawn on the merged image. ASIC1a shows a higher relative spine:shaft signal as compared to Lck-GFP, demonstrating that ASIC1a preferentially traffics to dendritic spines. *Data adapted from*[19,21] .

Besides the immunostaining results, biochemical analysis shows that ASIC1a and ASIC2a are enriched in brain synaptosomes [20,44,69]. Together, these data suggest an increased postsynaptic trafficking of ASICs. Consistent with this speculation, ASIC1a shows punctate staining along the dendrites in cultured brain neurons, which indicates that ASIC1a is clustered in dendritic spines (Figure 1A). To obtain high resolution images and study ASIC localization in a more physiological environment, we transfected epitope tagged ASIC1a and ASIC2a into organotypic hippocampal slices. Both ASIC1a and ASIC2a are present in most dendritic spines in slice neurons [19-21,71]. In addition, the relative level of ASIC1a in spines is higher as compared to that of a membrane-targeted Lck-GFP, demonstrating that ASIC1a is enriched in dendritic spines (Figure 1B). These data indicate a preferential trafficking of ASIC1a and ASIC2a to the postsynaptic membrane.

**Figure 2:**
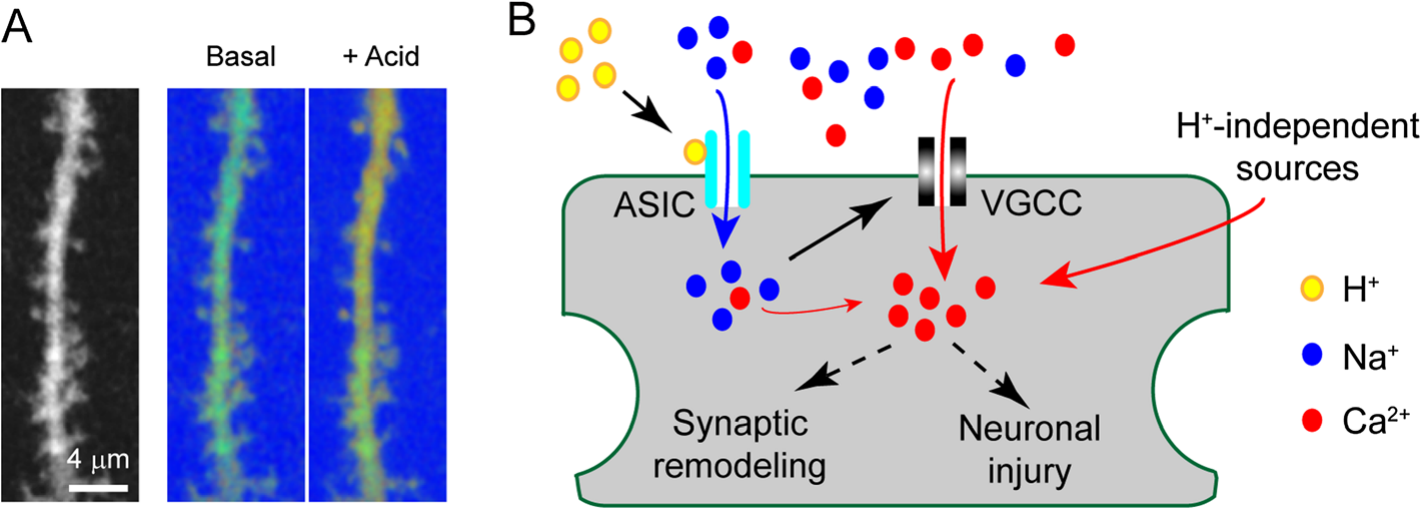
**Mechanisms by which protons increase [Ca**^**2+**^**]**_**i**_**. A**) Ca^2+^ imaging showing acidosis-induced [Ca^2+^_i_ increase in dendrites and dendritic spines. Organotypic hippocampal slices were transfected with cameleon and stimulated with a pH 6.0 solution. Left image shows the overview of the imaged dendritic segment. Right ratiometric images show YFP/CFP ratio (red indicates high while blue indicates low Ca^2+^) before and during acidic stimulation. Note the increase in Ca^2+^ in the dendrite and most dendritic spines. *Data adapted from*[20]*.***B**) Drawing showing how protons lead to [Ca^2+^_i_ increase in neurons. See text for detailed explanation. Depending on the magnitude and duration of the Ca^2+^ rise, acidosis can alter neural plasticity or, when [Ca^2+^_i_ passes certain threshold level, lead to neuronal injury.

Synaptic and dendritic targeting of ASIC1a is further supported by electrophysiological recordings and Ca^2+^ imaging experiments. Puffing acidic solutions to dendritic region elicits typical ASIC-type currents in hippocampal slice neurons [47]. In addition, using cameleon as a Ca^2+^ sensor, we have imaged acid-induced Ca^2+^ changes in hippocampal slices [19,20]. Acidic stimulation leads to a rapid increase of intracellular Ca^2+^ concentration ([Ca^2+^_i_) in dendritic spines, dendrites and cell bodies of pyramidal neurons (Figure 2A). The response is abolished in *ASIC1a−/−* slice neurons and reduced in *ASIC2−/−* slice neurons. These functional results, together with the immunolocalization and biochemical data, demonstrate that endogenous ASICs are not only preferentially trafficked to dendrites and spines but also function as the postsynaptic proton receptor there.

### ASIC and neuron physiology

#### Mechanisms by which ASIC activation increases [Ca^2+^]_i_

As stated above, ASIC activation mediates acidosis-induced [Ca^2+^_i_ rise in neurons. Part of this Ca^2+^ increase is due to direct Ca^2+^ entry through ASIC1a containing channels [73]. However, the relative permeability of Ca^2+^ is low: *p*Na^+^/*p*Ca^2+^ is 2.5, 18.5 and 25 in three studies on ASIC1a homomers [5,7,8], and ~4.1 for ASIC1a/2b heteromers [9]. Consistent with these data, several studies show that most of acidosis-induced [Ca^2+^_i_ increase in neurons are likely due to secondary mechanisms. In hippocampal slices, replacing extracellular Na^+^ with NMDG reduced acidic stimulation-induced [Ca^2+^_i_ rise by ~86%, showing that Na^+^ influx through ASICs is important [19]. In chick dorsal root ganglion neurons, direct Ca^2+^ entry through ASICs also contributes little to acid-induced Ca^2+^ increase [74]. Moreover, clamping the membrane potential at −70 mV abolishes acidosis-induced [Ca^2+^_i_ increase in cortical neurons [75], and inhibiting voltage-gated Ca^2+^ channels (VGCCs) largely eliminates acidosis-induced Ca^2+^ increase [19,75].

These data suggest the following model (Figure 2B): acidosis activates ASIC channels; Na^+^ along with a small number of Ca^2+^ enters the cell, which lead to membrane depolarization; then VGCCs get activated and initiate the main Ca^2+^ influx. This conclusion is important for understanding how ASICs impact neuron physiology. It suggests that non-calcium permeable ASIC channels, e.g., ASIC1a/2a heteromers, can make a significant contribution to acidosis-induced [Ca^2+^_i_ responses. Supporting this conclusion, deleting the *ASIC2* gene, which takes away both ASIC1a/2a and ASIC1a/2b heteromers, significantly reduces acidosis-induced Ca^2+^ rise [20]. This model also argues that when considering the effect of acidosis, the number of functional ASIC channels is important as opposed to the number of “Ca^2+^-conducting” populations.

Other downstream molecules are also involved in response to acidosis and ASIC activation. Besides VGCCs, store-operated release and sigma receptors also contribute to H^+^-induced [Ca^2+^_i_ increase in hippocampal slice neurons and dissociated cortical neurons, respectively [19,75]. CaMKII phosphorylation is also affected by altered ASIC1a expression. Brains isolated from ASIC1a null mice show a reduced CaMKII phosphorylation while those from the transgenic mice overexpressing ASIC1a have increased CaMKII phosphorylation [19]. This result suggests that manipulating ASIC1a expression level, and presumably ASIC activity, is sufficient to regulate Ca^2+^ signaling *in vivo*. How ASICs get activated in physiological conditions remains unclear. However, with functional magnetic resonance imaging, one recent report shows that learning induces acidification in human brain [76]. Although the exact magnitude of pH reduction in this paradigm remains to be determined, this study provides a direct support for potential ASIC activation by protons in physiological conditions.

#### ASIC and synaptic function

Their presence in dendritic spines suggests that ASICs regulate synaptic physiology. Several studies have tested this hypothesis in various systems. In hippocampal neurons, deleting the *ASIC1a* gene has no effect on basal levels of GABA, AMPA and NMDA currents [44,77]. However, *ASIC1a* deletion decreases the ratio of AMPA: NMDA currents in microisland hippocampal cultures [77]. Also in the microisland culture system, deleting the *ASIC1a* gene reduces paired-pulse responses. This result is consistent with a reduced long term potentiation in the ASIC1a knockout [44], and suggests that activation of ASICs contributes to increased synaptic efficacy in these conditions. One intriguing finding is that deleting the *ASIC1a* gene or inhibiting ASIC1a increases the frequency of miniature excitatory postsynaptic potentials in the microisland cultured neurons [77]. The mechanism for this observation remains unclear, but may indicate a possible feedback regulation as a consequence of reduced ASIC activity.

The exact role of ASIC1a at synapses may also depend on the experimental system studied. In contrast to the results in slices and cultured hippocampal neurons, inhibiting ASIC1a or silencing ASIC1a with siRNA in retina reduces photopic (cone-mediated) electroretinogram (ERG), but had little effect on scotopic (rod-mediated) ERG [78]. Deleting the *ASIC2* gene, on the other hand, increases scotopic ERG in response to mid- to high-intensity light, but has no effect on photopic ERG [52]. Despite the differential contribution of ASIC1a and ASIC2 in the rod and cone pathways, ASIC activation in both cases appears to be inhibitory on neurotransmission.

These electrophysiological findings are consistent with the synaptic localization of ASICs, and indicate that ASIC activation affects synaptic physiology. One pivotal question here is what is the contribution of ASICs to synaptic transmission, either in physiological or pathophysiological conditions. However, attempts to record an ASIC-initiated current during synaptic transmission have been unsuccessful [57]. This could be due to technical reasons. For example, H^+^ are buffered fast, which may impose difficulty for detecting small ASIC-generated synaptic currents when record at the cell body. The rapid desensitization of ASICs also adds technical challenges for detecting an ASIC specific contribution. Interestingly, one recent study shows that high frequency acidic stimulation can abolish the desensitization of ASIC1a, suggesting prolonged ASIC activation under similar conditions [79]. This result raises the possibility that, with further tweaking of the experimental protocol, along with the novel tools recently developed (e.g. the specific pharmacological reagents), one may uncover a specific contribution of ASICs to neurotransmission.

#### ASICs, acidosis and spine remodeling

In addition to the effects on synaptic physiology, ASICs also regulate structural remodeling of synaptic sites. In hippocampal slice neurons, both transient and chronic overexpression of human ASIC1a increases the number and length of dendritic spines [19]. Conversely, knockdown of ASIC1a with siRNA or expressing a dominant-negative ASIC1a construct reduces spine numbers. Deleting the *ASIC2* gene, which decreases synaptic targeting of ASIC1a, reduces the density of spine synapses [20]. These results show a good correlation between ASIC1a expression/synaptic levels and spine density. However, deleting the *ASIC1a* gene has no significant effect on dendritic spines [19]. The reason for this result is unclear. One possible, though not easy to test, explanation is that developmental compensation in the chronic knockout model leads to the lack of an effect in *ASIC1a* null slices. Another somewhat controversial finding is that overexpressing a mouse ASIC1a has no significant effect on spine number or length [21], which contrasts with the increased spine density observed with human ASIC1a overexpression. It is possible that the intrinsic difference between human and mouse ASIC1a is the cause. Supporting this speculation, human ASIC1a shows larger acid-activated current and is less susceptible to steady-state desensitization when compared to mouse ASIC1a [21,40]. Another possible contributing factor is the age of the cultures. In Jing et al. [21], slices were studied at the age equivalent to 17 days old, which is more mature than the 13 day old culture in Zha et al. [19]. In the literature, there are precedents that the effect on spines depends upon the developmental stages. One such example is A kinase anchor protein 150 (AKAP150). Disrupting the AKAP150 gene increases spine density at 2 weeks of age but this effect is not seen in adult stage [80]. Further study is needed to test these possibilities. Nevertheless, current data demonstrate that ASICs play an essential role in spine morphogenesis and/or maintenance.

The results above further suggest that acidosis, through activating ASICs, regulates spines/synapses. Indeed, treating organotypic hippocampal slices with a pH 6 buffer reduces the density and length of dendritic spines in a time-dependent manner [21]. As expected, deleting the *ASIC1a* gene abolishes acidosis-induced spine loss. These data reveal that, in addition to the “canonical” neurotoxic effect (see next section), acidosis also modulates synaptic remodeling.

Given the fact that acidosis typically lasts for some time in diseases, the above finding suggests that acidosis contributes to the change in brain plasticity in pathological conditions. However, several important questions remain to be answered. First, in Jing et al., spine remodeling was induced by 1 hr incubation in medium buffered at pH 6 [21]. Although a pH reduction to 6.0 or even lower occurs in conditions like severe ischemia, the duration in those cases is probably shorter, about 15–30 minutes [81]. However, a pH reduction to ~ pH 6.5 lasted for hours following middle cerebral artery occlusion (MCAO) [82]. Therefore, it is necessary to answer whether a persistent milder acidosis affects spines. It will also be interesting to reveal the effect of acute acidosis on spine dynamics. Timelapse experiments *in vitro* and *in vivo* will be valuable in addressing these questions. In addition, it will be important to ask whether acidosis contributes to spine remodeling in diseases, e.g., ischemia or seizures, which are known to reduce dendritic spines [83,84]. In parallel with the changes in spine morphology, it will also be important to ask what effect acidosis has on synaptic physiology, including plasticity. Answers to these questions will be pivotal for our understanding of how acidosis and ASICs regulate synaptic plasticity in diseases.

#### ASIC activation and neuronal injury

Through increasing [Ca^2+^_i_, acidosis can lead to neuronal injury (Figure 2B). This mechanism apparently contributes to a major part of ischemia-induced neuronal death [73,82,85,86]. In the MCAO model of brain ischemia, deleting the *ASIC1a* gene reduces MCAO-induced infarct volume [42,73,82]. Besides ischemia, deleting *ASIC1a* also slows down the disease progression in an experimental autoimmune encephalomyelitis model [54]. These data indicate that ASIC1a activation exacerbates neuronal injury in certain disease paradigms. ASIC activation can also be beneficial. For example, the activation of ASIC1a is required for attenuating seizure progression; deleting the *ASIC1a* gene or inhibiting ASIC1a increases seizure severity [87]. Similar mechanism may also apply to other systems. In retina, deleting the *ASIC2* gene increases the susceptibility of retina neurons to bright illumination-induced damage [52]. In *ASIC2−/−* but not wild-type retina, the outer and inner nuclear layers, ganglion cell layer, and pigmented epithelium all show increased apoptosis following a two-hour challenge with bright light.

The results on seizure and light-induced retina damage appear counterintuitive at the first sight, because ASIC activation leads to increased [Ca^2+^_i_ and neuronal firing, which typically leads to excitotoxicity. However, ASICs are expressed in both excitatory and inhibitory neurons. As a matter of fact, many interneurons exhibit higher ASIC currents as compared to pyramidal neurons [47,87,88]. Therefore, through differential activation of excitatory or inhibitory neurons, acidosis can increase or attenuate the excitability of the whole network. This model will be an interesting one to test in future studies. However, both inhibitory and excitatory neurons show mixed expression of ASIC1a homomeric and ASIC1a/2a heteromeric channels [47], suggesting that selective pharmacological interventions to inhibit only interneurons or only pyramidal neurons will be difficult. One possible approach is to genetically delete ASICs in either inhibitory or excitatory neurons, and assess the outcome in various neurological diseases.

### Molecular mechanisms regulating ASIC trafficking and function

#### ASIC1a trafficking on neuronal injury and spine remodeling

Increased ASIC1a surface trafficking or channel activity potentiates acidosis-induced neuronal death [41,42] and leads to hyperalgesia in response to mechanical stimulation [89]. These results indicate that the forward trafficking of ASICs is critical for acidosis-induced neuronal injury. Besides surface expression, trafficking to dendritic regions may also be important for acidosis-induced changes. An ASIC1a mutant with increased dendritic and surface levels potentiates acidosis-induced spine loss [21]. Conversely, an ASIC1a mutant with reduced trafficking has the opposite effect. Since both mutants affect both surface levels and dendritic targeting, it is difficult to conclude whether the effect observed is due to dendritic ASIC1a specifically. Nevertheless, these data support the speculation that dendritically targeted ASIC1a (as opposed to those at the cell body) mediates the effect of acidosis on spine remodeling.

The above data on ASIC trafficking are based on static images/samples before and after treatment. To better understand the mechanism, it will be important to visualize real-time movement of ASICs following interventions. For example, how do changes in neural activity affect ASIC trafficking, either to and from cell surface, or to synaptic sites? How do disease conditions, e.g., increased ROS production, regulate ASIC trafficking in live neurons? To address these questions, specific antibodies recognizing extracellular domain of ASICs are needed. Alternatively, one may introduce extracellularly tagged ASICs into neurons. However, it is important to note that, probably due to the complexity of its extracellular domain, inserting extracellular tags typically impairs the trafficking of ASICs ([90] and our own experience).

#### N-glycosylation regulates ASIC trafficking and function

As one step toward understanding ASIC trafficking, recent studies have assessed the role of *N*-glycosylation in ASIC biogenesis. In both heterologous cells and brain slices, surface ASIC1a contains a higher percentage of EndoH-resistant population, which indicates that their *N*-linked glycans have been further processed (or “matured”) in mid-late Golgi [21,71]. Although the maturation of *N*-linked glycans is not required for ASIC1a surface expression, matured ASIC1a apparently traffics preferentially to the cell surface. Tunicamycin, which inhibits the addition of *N*-linked sugars [91], reduces ASIC1a surface levels [21]. In addition, tunicamycin decreases dendritic targeting of ASIC1a in hippocampal slices, and reduces both the amplitude and desensitization rate of ASIC1a current in CHO cells. These data indicate that the maturation of *N*-glycans on ASICs is important for their proper spatial distribution and channel function.

The number of potential *N*-glycosylation sites in different ASIC subunits ranges from two to four, with ASIC1a and 2a each contains two. Both of the two potential glycosylation sites on ASIC1a and ASIC2a are glycosylated in heterologous cells [71,90,92]. Two separate studies have assessed the effect of glycosylation mutants on ASIC1a channel properties [71,90]. Mutating the Asn393 site reduces ASIC1a maturation and acid-activated current. One note to make is that the Asn393 site, which locates between the α6 and α7 helices in the crystal structure, is conserved in all ASICs [4]. It will be interesting to ask whether the glycosylation of this site has similar effect in other subunits. Unlike Asn393, the effect of Asn366 mutation differs between the two published studies. In an earlier study, a rat N366A mutant reduces acid-activated current in *Xenopus* oocytes [90]. In contrast, we found that mutating the Asn366 site in either mouse or rat ASIC1a increases surface expression and pH-activated current in both CHO cells and *Xenopus* oocytes [71]. In our study, the mouse N366Q mutant also shows increased dendritic targeting in hippocampal slices, and when compared to WT ASIC1a, N366Q further potentiates acidosis-induced spine loss; both observations are consistent with an increased trafficking of the N366Q mutant. While the reason for the discrepancy between the two studies is not obvious, it is clear that *N*-glycosylation is important for ASIC1a regulation.

These data further imply that processes controlling protein maturation through the secretory pathway play critical roles in regulating ASIC function. Consistent with this speculation, one report shows that most ASIC1a is trapped in endoplasmic reticulum (ER) but undergoes rapid membrane insertion in response to serum deprivation [68]. In addition, glycerol, a common reagent that facilitates protein maturation, increases ASIC2 surface expression in vascular smooth muscle cells [93]. Similar mechanisms may also regulate ASIC biogenesis in disease conditions. In one study, MCAO reduces ASIC1a expression levels within 30 minutes [94]. Although it remains unclear whether ASIC1a trafficking is affected in this paradigm, the result suggests that altered ASIC biogenesis regulates ischemia-induced neuronal injury. In addition, increased oxidation may also regulate ASIC trafficking. H_2_O_2_, a reactive oxygen species (ROS), reduces ASIC1a surface expression [95]. It will be interesting to see whether factors regulating ER stress and/or ROS production regulate ASIC1a surface and dendritic trafficking, and to ask how proteins that control the secretory process affect ASIC trafficking and function.

#### Motifs within ASICs

All ASICs contain a C-terminal *PDZ binding motif*. The C-termini of ASIC1 and ASIC2 are DFTC and EIAC, respectively, and more closely resemble that of a type II motif [67,96,97]. In contrast, the last four amino acid of ASIC3, VTRL, fits a canonical type I motif [98]. Through the interaction with several PDZ proteins, these motifs regulate ASIC surface expression, synaptic targeting, and channel function (see next section). Another motif identified within the C-terminus of ASIC1a is a LDDVK motif, which functions as an *α-actinin interacting motif*[99]. Mutating this motif has no apparent effect on ASIC1a surface expression, but slight decreases the pH sensitivity of ASIC1a. At the N-terminus, all ASICs contain a *His-Gly (HG) motif*, which is a signature motif for EDA family ion channels. In ENaCs, mutating the HG motif reduces the open probability of these channels [100,101], suggesting its importance in channel gating. The functional significance of the HG motif in ASICs, however, remains unclear. Other than these three examples, we know little about motifs in ASICs. The characterization of novel motifs within ASICs will help us to understand molecular mechanisms regulating these channels, facilitate the identification of new ASIC interacting proteins, and also provide potential sites for intervening acidosis-induced changes.

#### Proteins regulating ASICs

Through direct association or protein modification, effector proteins and/or signaling molecules regulate the function of various ion channels. The number of ASIC-regulators has kept increasing in the past few years. A summary of these effector proteins and signaling molecules is listed below.

#### Effector proteins

##### α-Actinin

α-Actinin is one family of molecules important for linking the actin cytoskeleton with the synaptic complex. Actinin-1 and −4 interact with ASIC1a through a C-terminal LDDVK motif [99]. In heterologous cells, the interaction with actinin-4 but not that with actinin-1 regulates ASIC1a current properties; pH sensitivity and recovery from desensitization are increased with actinin-4 overexpression. In hippocampal neurons, knocking down actinin 1, 2 and 4 together increases ASIC current density and reduces pH_50_[99].

##### AKAP150

AKAP150 is the major AKAP for anchoring PKA to synapses [102]. AKAP150 associates and co-localizes with ASIC1a and ASIC2a, and increases ASIC1a current [70]. The effect on current is likely due to a PKA-dependent mechanism. Ht-31, a small peptide that inhibits the association between PKA and AKAP150, reduces acid-activated current in cultured cortical neurons [70].

##### Annexin II light chain/p11

p11 is an adaptor protein which forms a complex with Annexin II [103]. p11/Annexin II complex interacts with multiple ion channels and regulates their trafficking. p11 interacts with the N-terminus of ASIC1a but not with other ASICs [104]. Through this interaction, p11 increases ASIC1a surface expression and current density without changing other properties of the current.

##### Chaperones

Several chaperone proteins interact with ASICs with different specificity. Hsp90 and Grp4 interact with ASIC1a, Hsc70 interacts with ASIC2a, Grp78 and calnexin interact with both ASIC1a and 2a [105]. Knocking down Hsc70 increases ASIC2a surface expression in both glioma and muscle cells [93,105]. The detailed molecular mechanism for how chaperones affect ASIC trafficking and function remains to be clarified.

##### CIPP (channel-interacting PDZ domain protein)

CIPP contains four PDZ domains. Through its fourth PDZ, CIPP interacts with ASIC3 and increases its current density [106]. This effect is most likely due to an increase in the number of functional channels because CIPP appears to have no effect on open probability or single channel conductance of ASIC3.

##### Lin-7b

Lin-7b associates with ASIC3 in COS cells [98]. This interaction increases surface expression and current amplitude of ASIC3.

##### MAGI-1b

MAGI-1b is a member of the MAGUK (membrane-associated guanylate kinases) family. MAGI-1b co-immunoprecipitates with ASIC3 in COS cells [98]. The functional effect of MAGI-1b on ASIC3 remains unclear.

##### NHERF (Na+/H^+^ exchanger regulatory factor)

Both NHERF1 and NHERF2 interact with ASIC3 via PDZ dependent interactions [107]. In COS cells, NHERF1 alters the distribution of ASIC3, which becomes colocalized with the ezrin/radixin/moesin family proteins. The interaction with NHERF1 increases surface expression, peak amplitude and sustained current of ASIC3. In addition, PKC enhances the association between NHERF1 and ASIC3 through the phosphorylation of serine 523 on ASIC3.

##### PICK1 (Protein-interacting with C-kinase)

PICK1 is a scaffolding protein that regulates the trafficking of multiple synaptic receptors [108]. PICK1 interacts with ASIC1a and ASIC2 through the PDZ-dependent interactions [67,96]. The interaction between PICK1 and ASIC1a increases surface expression of ASIC1a, and this effect depends upon the BAR domain and lipid binding of PICK1 [109,110]. The interaction between ASIC1a and PICK1 is regulated by protein kinase A (PKA) and protein kinase C (PKC) (see below).

##### PIST

PIST is a PDZ containing protein that was originally identified as an interactor with TC10, a Rho GTPase[111]. PIST was identified as an ASIC3 interactor in a two-hybrid screening. The functional effect of PIST on ASIC3 is unknown [98].

##### PSD-95 (Postsynaptic density protein 95)

PSD-95 is the primary organizer of the postsynaptic density complex. Through PDZ-dependent interactions, PSD-95 associates with ASIC2a and ASIC3 but not ASIC1a [20,98]. In heterologous cells, the interaction between PSD-95 and ASIC3 occurs in lipid rafts, and reduces surface expression and current amplitude of ASIC3 [98,112]. In hippocampal slices, PSD-95 is important for synaptic targeting of ASIC2a, which in turn increases the synaptic levels of ASIC1a and acidosis-induced [Ca^2+^_i_ responses in spines [20].

##### Stomatin and Stomatin-like protein 3 (SLP3)

Stomatin and SLP3 co-immunoprecipitate with ASIC1a, 2a and 3 in heterologous cells [113-115]. Dimerization of stomatin is essential for its interaction with ASICs [116]. Stomatin has no effect on ASIC1a current, increases the rate of desensitization of ASIC2a, and drastically reduces ASIC3 current. SLP3, which forms a complex with both stomatin and ASIC2a, reduces ASIC2a current without affecting its surface expression. In the SLP3 knockout mice, ASIC current is increased in parallel with a disruption of touch sensation.

#### Signaling molecules regulating ASICs

##### Ca^2+^/calmodulin-dependent protein kinase II (CaMKII)

The C-terminus of ASIC1a contains a KRXS sequence, which can be phosphorylated by CaMKII and PKA. CaMKII phosphorylation of ASIC1a increases its current amplitude and probably contributes to ischemia-induced neuronal death [86]. This finding also suggests that conditions which alter [Ca^2+^_i_ levels play an active role in regulating ASIC1a and consequently acidosis-related processes. Interestingly, acidosis increases [Ca^2+^_i_ via ASIC1a, and manipulating ASIC1a expression levels regulates CaMKII phosphorylation [19]. These findings suggest a possible positive feedback mechanism contributing to acidosis-induced changes in neural plasticity and excitotoxicity.

##### Protein kinase A (PKA)

PKA phosphorylates ASIC1a at the same site as CaMKII. PKA phosphorylation of ASIC1a inhibits its association with PICK1 and reduces the clustering of ASIC1a inside the cell [117]. Further study is needed to clarify how PKA regulates ASIC function in neurons, and whether PKA and CaMKII share similar mechanisms in regulating acidosis-induced changes.

##### Phosphoinositide 3-kinase (PI3K)

Activation of PI3K by BDNF results in increased surface expression of ASIC1a and ASIC2a, but not that of ASIC1b or ASIC3 [89]. This effect was attributed to increased insertion as opposed to a reduction in endocytosis of ASIC1a. The serine 25 site in ASIC1a is important for the observed effect. An ASIC1a S25A mutant has reduced surface expression and abolishes BDNF-induced increase in current. Ser25 phosphorylation apparently contributes to part of brain derived neurotrophic factor (BDNF)-induced hypersensitivity to painful stimuli. These results establish a connection between neutrophin signaling and ASIC regulation. One remaining question is which kinase directly phosphorylates the Ser25 site in ASIC1a, which does not appear to fit the consensus phosphorylation sites of the common kinases.

##### Insulin signaling

Insulin deprivation increases ASIC1a surface trafficking and potentiates acid-activated currents [68]. Insulin initiates multiple downstream signaling events, including the activation of PI3K/Akt pathway [118]. However, since PI3K activation leads to increased ASIC1a trafficking [89], the effect of insulin is most likely mediated by an PI3K-independent pathway.

##### Protein kinase C (PKC)

PKC regulates both ASIC1a and ASIC2a. In *Xenopus* oocytes, PKC activation reduces current amplitude of human ASIC1a [119]. Serine 40 and 499 appear to be involved. An S40E mutant shows reduced current (mimics the effect of PKC), while an S40A mutant abolishes the effect of PKC activators. Unlike serine 40, both S499A and S499E show reduced current amplitude, and S499A does not abolish the effect of PKC activation. These effects on currents are unlikely a result of reduced expression, because all mutants show similar protein levels in the total membrane fraction. However, it remains to see whether the surface trafficking of ASIC1a is affected by PKC. In contrast to the findings in oocytes, PKC increases ASIC current in cultured cortical neurons [110]. This effect may due to ASIC2. In COS cells, PKC activation increases ASIC2a current [120]. Threonine 39 in ASIC2a, the equivalent of serine 40 in ASIC1a, is important for the effect of PKC on ASIC2a. In addition, the potentiation of ASIC current by PKC depends upon an interaction with PICK1.

##### Reactive oxygen species (ROS)

Many diseases increase ROS production. Since ASICs contain a large number of cysteines, several studies have studied whether redox reagents affect ASIC channel properties. Reducing reagents increase ASIC1a current while oxidizing reagents have the opposite effect [88,121,122]. These effects are largely due to modifications in the extracellular domain. In contrast to oxidizing reagents, nitric oxide (NO) potentiates ASIC1a, -1b, -2a, and −3 through the modulation of extracellular domain [123]. In addition, ROS can target intracellular C-terminal cysteines. H_2_O_2_ induces the formation of intersubunit disulfide bonds between C-terminal cysteines within ASIC1a, and reduces its surface expression [95].

### Summary and speculations

Research in recent years has demonstrated that acidosis and ASICs are an important contributor to multiple neurological diseases. To better understand the role of ASICs in diseases, it is now necessary to answer some fundamental questions regarding the regulation and function of ASICs. A few of these questions are discussed in this review and/or summarized below:

What is the contribution of ASICs to synaptic transmission?

What is the dynamics of acidosis-induced synaptic remodeling?

How do acidosis and ASICs contribute to synaptic plasticity in diseases?

Are there endogenous ASIC ligands other than protons?

*How does the exact channel stoichiometry affect ASIC function?* For exmaple, are channels containing 1 ASIC1a and 2 ASIC2a subunits behave the same as channels containing 2 ASIC1a and 1 ASIC2a? Or do the heteromeric channels prefer one stoichiometry over another?

What is the role of altered ASIC trafficking in synaptic remodeling and diseases?

What novel motifs and accessory proteins contribute to ASIC regulation?

What signaling pathways regulate ASIC trafficking and function?

The list apparently can go on longer. However, these are some of the basic ones on ASIC biology. Answering these questions will bring novel insight into the regulation of ASICs and their role in neuron physiology, and will lay the groundwork for targeting these ion channels in diseases.

## Abbreviations

AMPA: 2-amino-3-(3-hydroxy-5-methyl-isoxazol-4-yl)propanoic acid; ASIC: Acid-sensing ion channel; CaMKII: Ca^2+^/calmodulin-dependent protein kinase II; CHO: Chinese hamster ovary; DEG: Degenerin; EDA: ENaC/DEG/ASIC/; ENaC: Epithelial sodium channels; ER: Endoplasmic reticulum; ERG: Electroretinogram; MCAO: Middle cerebral artery occlusion; MAGI-1b: (membrane-associated guanylate kinases) family; NHERF: Na^+^/H^+^ exchanger regulatory factor; NMDA: N-Methyl-D-aspartic acid; PDZ: PSD-95/disc large tumor suppressor/zonula occludens-1; PI3K: Phosphoinositide 3-kinase; PICK1: Protein-interacting with C kinase; PKA: Protein kinase A; PKC: Protein kinase C; PSD-95: Postsynaptic density protein 95; ROS: Reactive oxygen species; SLP3: Stomatin-like protein 3; VGCC: Voltage gated calcium channel.

## Competing interest

The author declare that he has no conflict of interest.
